# Stonin1 mediates endocytosis of the proteoglycan NG2 and regulates focal adhesion dynamics and cell motility

**DOI:** 10.1038/ncomms9535

**Published:** 2015-10-05

**Authors:** Fabian Feutlinske, Marietta Browarski, Min-Chi Ku, Philipp Trnka, Sonia Waiczies, Thoralf Niendorf, William B. Stallcup, Rainer Glass, Eberhard Krause, Tanja Maritzen

**Affiliations:** 1 Leibniz-Institut für Molekulare Pharmakologie (FMP), Robert-Rössle-Strasse 10, 13125 Berlin, Germany; 2 Berlin Ultrahigh Field Facility (B.U.F.F.), Max-Delbrueck Center for Molecular Medicine, Robert-Rössle-Strasse 10, 13125 Berlin, Germany; 3 German Centre for Cardiovascular Research (DZHK), Partner Site, Robert-Rössle-Strasse 10, 13125 Berlin, Germany; 4 Tumor Microenvironment and Metastasis Program, Cancer Center, Sanford-Burnham Medical Research Institute, 10901 North Torrey Pines Road, La Jolla, California 92037, USA; 5 Neurosurgical Research, Clinic for Neurosurgery, Ludwig Maximilians University of Munich, Marchionistrasse 15, 81377 Munich, Germany

## Abstract

Cellular functions, ranging from focal adhesion (FA) dynamics and cell motility to tumour growth, are orchestrated by signals cells receive from outside via cell surface receptors. Signalling is fine-tuned by the exo–endocytic cycling of these receptors to control cellular responses such as FA dynamics, which determine cell motility. How precisely endocytosis regulates turnover of the various cell surface receptors remains unclear. Here we identify Stonin1, an endocytic adaptor of unknown function, as a regulator of FA dynamics and cell motility, and demonstrate that it facilitates the internalization of the oncogenic proteoglycan NG2, a co-receptor of integrins and platelet-derived growth factor receptor. Embryonic fibroblasts obtained from Stonin1-deficient mice display a marked surface accumulation of NG2, increased cellular signalling and defective FA disassembly as well as altered cellular motility. These data establish Stonin1 as a specific adaptor for the endocytosis of NG2 and as an important factor for FA dynamics and cell migration.

## Abstract (web summary)

Signalling is often fine-tuned by the exo-endocytic cycling of cell surface receptors. Here, the authors show that the endocytic adaptor protein Stonin1 is important for the endocytosis of NG2, a co-receptor for extracellular matrix and growth factors, and that loss of Stonin1 alters cell motility.

## Graphical abstract

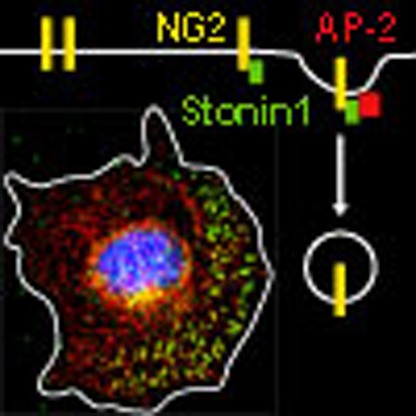

Endocytosis is essential for the regulation of cellular signalling by adjusting the number and localization of receptors at the cell surface. Alterations in endocytosis affect focal adhesion (FA) dynamics and cellular migration, which crucially depend on the precise spatiotemporal regulation of the surface levels of various adhesion proteins^1^. In fact, dysregulated endocytosis causes defects in cellular motility^1^ and is associated with cancer^2^.

FAs represent complex contact sites between cells and extracellular matrix that are especially rich in cell surface receptors to mediate cellular adhesion and signalling^3^. While integrins are the best-studied adhesion receptors within FAs, adhesion sites contain numerous additional cell surface proteins such as syndecans and other proteoglycans. Although it is clear that dynamic FA turnover is essential for cell motility and requires exo- and endocytosis, we still lack detailed knowledge about how the turnover of the various FA components is controlled. While FA disassembly was shown to rely on clathrin^4^,^5^ and the cargo-specific adaptors ARH and Dab2 (ref. 5) to mediate integrin uptake, the fate of other FA components remains unclear.

Cargo-specific adaptors are required whenever surface proteins do not contain the necessary consensus motifs to bind to the general endocytic adaptor AP-2, which recruits most cargo proteins for clathrin-mediated endocytosis^6^. By linking their corresponding cargo to the endocytic machinery, they ensure its efficient internalization. The group of cargo-specific adaptors includes also the Stonin proteins, which interact with AP-2 and cargo proteins^7^. While *Drosophila melanogaster* contains a single Stonin protein^7^ termed StonedB, homology searches in mammals led to the cloning of two Stonin orthologues^8^. Stonin2 proved functionally similar to StonedB and acts as an endocytic adaptor for the synaptic vesicle protein Synaptotagmin1 (ref. 9). In contrast, the role of Stonin1, the other mammalian orthologue, remained enigmatic. The fact that combined loss of Stonin1 and Stonin2 does not aggravate the neuronal defects of Stonin2-deficient mice^9^ suggests that Stonin1 serves an unknown function distinct from that of Stonin2.

In this study, we deleted Stonin1 in mice and found that it acts as an important regulator of FA dynamics and cellular motility. We show that Stonin1 is crucial for the efficient internalization of the proteoglycan NG2 (also known as CSPG4, AN2, MCSP and HMP), an FA-associated transmembrane protein serving as a co-receptor for integrins and the platelet-derived growth factor receptor (PDGFR)^10^, and as a promoter of cellular motility^11^,^12^,^13^,^14^,^15^,^16^,^17^ and tumour growth^18^,^19^,^20^. In the absence of Stonin1, NG2 accumulates at the cell surface, which alters cell migration. Thus, we establish Stonin1 as a specific endocytic adaptor for NG2 with important roles in FA dynamics and cellular motility.

## Results

### Stonin1 is an endocytic adaptor localizing close to FAs

**Figure 1:**
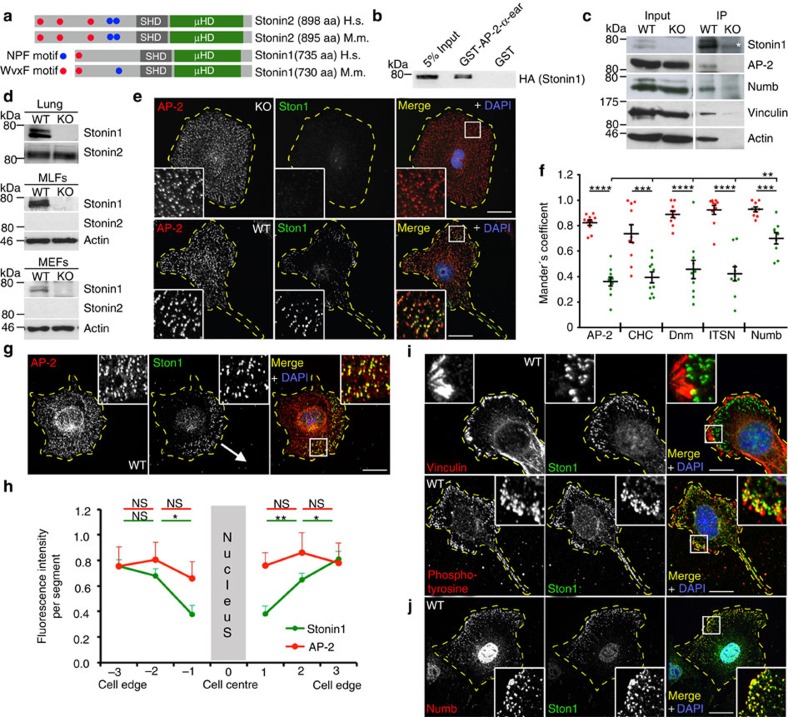
Stonin1 is an endocytic adaptor localizing close to FAs. (**a**) Stonin1 and Stonin2 domain structure. SHD, Stonin homology domain; μHD, μ-homology domain. H.s., *Homo sapiens*; M.m., *Mus musculus*. (**b**) Stonin1 interacts with AP-2. Bead-coupled GST-AP-2α-ear resp. GST as control was incubated with lysate from cells overexpressing Stonin1-haemagglutinin (HA). Bound proteins were eluted from washed beads and analysed by immunoblotting with HA-specific antibodies. (**c**) Stonin1 associates with endocytic and FA proteins. Bead-coupled Stonin1-specific antibodies were incubated with WT and *Stonin1*^*−/−*^ lung lysate. Bound proteins were eluted from washed beads and analysed by immunoblotting with the indicated antibodies (* indicates an unspecific band). (**d**) Stonin1 and Stonin2 protein expression in different tissue and cell types. Lysates from WT and *Stonin1*^*−/−*^ cells were analysed by immunoblotting using antibodies specific for Stonin1, Stonin2 and actin. MLFs, mouse lung fibroblasts; MEFs, mouse embryonic fibroblasts. (**e**–**j**) Stonin1 co-localizes with endocytic proteins at peripheral CCPs close to FAs. (**e**,**g**,**i**,**j**) Fixed WT and *Stonin1*^*−/−*^ MEFs were stained with antibodies specific for the indicated proteins and analysed by confocal microscopy. Insets show enlargements of boxed areas. DAPI-stained nuclei are depicted in blue. Scale bar, 25 μm. (**f**) Quantification of co-localization from images such as in **e** using Mander's coefficients. Red: correlation of Stonin1 with endocytic proteins; green: reverse correlation (data are depicted as mean±s.e.m., *n*=9–10, unpaired two-tailed Student's *t*-test for comparisons between two types of correlations for the same staining, one-way analysis of variance followed by Dunnett post-test to compare Mander's coefficients for correlation of Numb with Stonin1 with Mander's coefficients for correlations of other endocytic proteins with Stonin1, ****P<0.0001, ***P<0.001, **P<0.01). Only the outer 2/3 of cell area were evaluated to exclude unspecific nuclear background staining. (**h**) Comparison of Stonin1 and AP-2 distribution across the cell based on clock scans on images such as in **e** (data are depicted as mean±s.e.m., *n*=14, unpaired two-tailed Student's *t*-test, **P<0.01, *P<0.05). DAPI, 4,6-diamidino-2-phenylindole; NS, not significant.

Stonin1 and Stonin2 share WVxF motifs for interacting with AP-2, a central Stonin homology domain and a cargo-binding C-terminal μ-homology domain (μHD) (Fig. 1a). This suggests that Stonin1 functions as an endocytic adaptor for a so far unknown cargo. In line with this, Stonin1 binds to AP-2 in glutathione *S*-transferase (GST)-pull-down assays (Fig. 1b) and co-immunoprecipitations (Fig. 1c). To unravel the function of Stonin1, we created *Stonin1*^*−/−*^ (knockout, KO) mice (Supplementary Fig. 1) and derived mouse embryonic fibroblasts (MEFs) from them. In contrast to organs such as lung, fibroblasts express only Stonin1, but not Stonin2 (Fig. 1d), making them well-suited for studying Stonin1 function. Immunofluorescence stainings revealed a high degree of co-localization of Stonin1 with endocytic proteins such as AP-2, clathrin, dynamin2 and intersectin at clathrin-coated pits (CCPs), but not with the endosomal adaptor AP-1 (Fig. 1e,f; Supplementary Fig. 2a,b). However, while AP-2-positive CCPs are distributed evenly across the cell surface, Stonin1 localizes specifically to a subset of peripheral CCPs (Fig. 1g,h). In migrating cells, Stonin1-positive CCPs localize predominantly to the leading edge (Fig. 1g) and are found adjacent to FAs enriched in vinculin and phosphotyrosinated proteins (Fig. 1i). This characteristic staining pattern is shared by Numb, a specific adaptor for β1-integrin endocytosis at disassembling FAs^21^. Co-stainings with Stonin1- and Numb-specific antibodies revealed a significantly higher co-localization of Numb with Stonin1 than for any other endocytic protein (Fig. 1f,j) and, thus, suggested a role for Stonin1 in FA dynamics. The association of Stonin1 with FAs was corroborated by biochemical experiments. Stonin1 immunoprecipitations followed by immunoblotting revealed, in addition to endocytic proteins such as AP-2, FA components such as vinculin as binding partners (Fig. 1c). Thus, Stonin1 localizes to peripheral endocytic sites nearby FAs and interacts with FA components, suggesting that it may regulate FA turnover and/or dynamics.

### Stonin1 regulates FA dynamics

**Figure 2:**
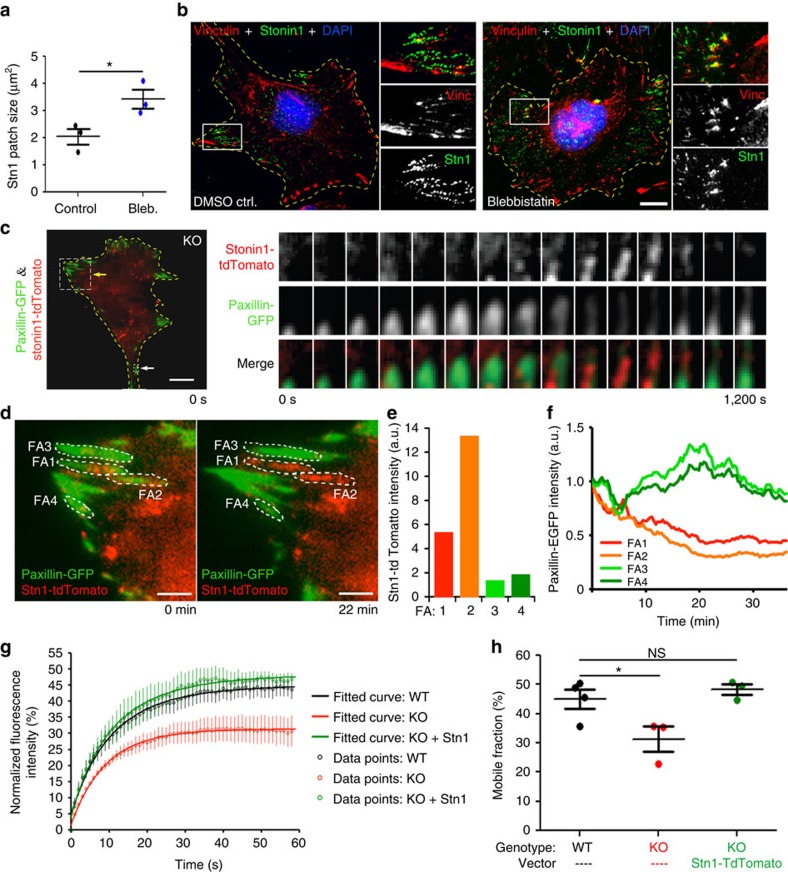
Stonin1 regulates FA dynamics. (**a,b**) Effect of blebbistatin on Stonin1 in MEFs. (**a**) Blebbistatin (50 μM for 30 min) induces larger Stonin1 patches. Quantification of absolute size of Stonin1-positive structures in control and blebbistatin-treated cells (data are depicted as mean±s.e.m., *N*=3, *n*=20–28 per experiment, unpaired two-tailed Student's *t*-test, *P<0.05). (**b**) Acute blebbistatin treatment (25 μM for 5 min) increases the co-localization of Stonin1 with the FA marker vinculin. Blebbistatin- or dimethylsulphoxide (DMSO)- (=control) treated cells were fixed and stained with vinculin- and Stonin1-specific antibodies. Scale bar, 25 μm. (**c**) Stonin1 accumulates at disassembling FAs. Images derived from live-cell TIRF time-lapse movie of *Stonin1*^*−/−*^ MEF transfected with Stonin1-tdTomato and Paxillin-GFP (60 min movie with 20-s intervals; pixels were smoothened by ImageJ). Left: overview of an imaged cell at the begining of the movie. Scale bar, 25 μm. Right: time series of the boxed area indicated with a white arrow. (**d**–**f**) High levels of Stonin1 correlate with FA disassembly, while low Stonin1 levels are found in stable FAs. (**d**) Enlargement of the boxed area indicated by a yellow arrow in **c** at 0 min (left) and 22 min (right) (see also Supplementary Movie 1). Scale bar, 6.25 μm. (**e**) Quantification of Stonin1-tdTomato fluorescence intensity per area (background subtracted) summarized over 35 min within leading edge FAs #1–4 as defined by Paxillin-EGFP signal (see outline). (**f**) Quantification of the Paxillin-EGFP fluorescence intensity over time as a measure for FA stability for FAs #1–4. (**g**,**h**) Slowed FA dynamics in the absence of Stonin1. (**g**) Curves depicting % fluorescence recovery after photobleaching (FRAP) for Paxillin-EGFP-transfected WT and *Stonin1*^*−/−*^ MEFs. Original data points±s.e.m. are shown (open circles) as well as fitted curves (solid lines). Re-expression of Stonin1-tdTomato rescued the decreased recovery in *Stonin1*^*−/−*^ MEFs. (**h**) Quantification of the recovered, that is, mobile fraction of Paxillin-EGFP molecules based on the exponential fit of the FRAP data (data are given as mean±s.e.m., *N*=4 for WT, *N*=3 for KO and rescue, one-way analysis of variance followed by Dunnett's post-test, *P<0.05). NS, not significant.

Consistent with our findings, two proteomic studies previously identified Stonin1 as a FA component^22^,^23^. Stonin1 was one of the few proteins not detected in steady-state FAs, but found selectively when FA disassembly was triggered by blebbistatin-mediated inhibition of myosinII^22^. MyosinII-dependent tensile forces are necessary for FA maturation and integrity^24^. Their loss prevents FA maturation and causes the disassembly of mature FAs^25^, resulting in an enrichment of nascent adhesions. To catch FAs in the process of disassembly, we treated cells briefly with blebbistatin. Under these conditions Stonin1 formed enlarged patches, which partially co-localized with vinculin and likely represent disassembling FAs (Fig. 2a,b), suggesting that Stonin1 preferentially localizes to disassembling FAs. To study this process in living cells, we performed dual-colour total internal reflection fluorescence (TIRF) imaging of *Stonin1*^*−/−*^ MEFs expressing tdTomato-Stonin1 and the FA marker enhanced green fluorescent protein (EGFP)-Paxillin. Stonin1 fluorescence accumulated precisely at the time when Paxillin fluorescence faded as FAs disassembled. Conversely, Stonin1 levels declined as new FAs formed (Fig. 2c). The fact that a fraction of FAs was stable during the time course of imaging while others progressively lost the Paxillin signal, provided us with a means to correlate FA behaviour with Stonin1 levels. Indeed, FAs with high Stonin1 levels typically underwent disassembly, whereas stable FAs usually lacked Stonin1 (Fig. 2d–f; Supplementary Movie 1).

**Figure 3:**
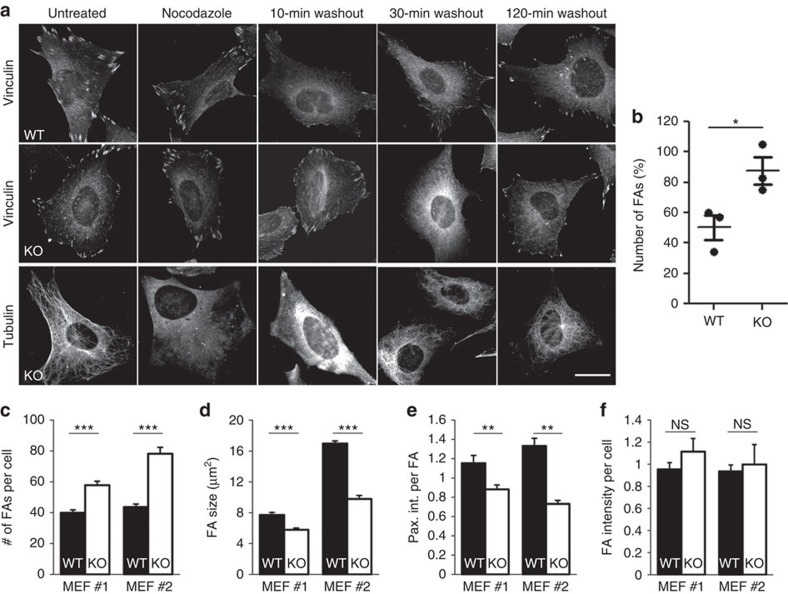
Stonin1 is required for FA disassembly. (**a,b**) Loss of Stonin1 slows FA disassembly. (**a**) Starved WT and *Stonin1*^*−/−*^ MEFs were left untreated or were incubated with 10 μM nocodazole. After 4 h, nocodazole was washed out to allow microtubule regrowth to trigger FA disassembly. Cells were fixed and immunostained with vinculin- and tubulin-specific antibodies. Scale bar, 25 μm. (**b**) The number of FAs in WT and *Stonin1*^*−/−*^ cells after 10 min of nocodazole washout relative to untreated cells was quantified by thresholded particle counting of vinculin-positive FAs (data are depicted as mean±s.e.m., *N*=3, *n*>100, unpaired two-tailed Student's *t*-test, *P<0.05). (**c**–**f**) Alterations in steady-state FA number and size upon Stonin1 deletion. Quantification of FA number per cell (**c**), FA size (**d**) Paxillin intensity per FA (**e**) and total Paxillin intensity per cell (**f**) in two independent WT and *Stonin1*^*−/−*^ MEFs lines fixed and stained with Paxillin-specific antibodies (data are depicted as mean±s.e.m., *N*=3, *n*=116, unpaired two-tailed Student's *t*-test, ***P<0.001, **P<0.01). NS, not significant.

To dissect whether Stonin1 modulates FA dynamics, we employed fluorescence recovery after photobleaching (FRAP). Previous FRAP studies demonstrated that FAs differ in their dynamic protein-exchange rate and in the density of their components, which affects FA remodelling^26^,^27^,^28^. Paxillin-EGFP-expressing wild-type (WT) and *Stonin1*^*−/−*^ MEFs were photobleached, and FRAP was measured. While the kinetics of recovery did not differ significantly between WT and *Stonin1*^*−/−*^ MEFs (*t*_1/2_(WT)=8.24±2.03 s, *N*=4; *t*_1/2_(KO)=6.20±1.04 s, *N*=3; data given as mean±s.e.m.; compare Fig. 2g), its extent was strongly reduced in *Stonin1*^*−/−*^ MEFs (Fig. 2g,h; Supplementary Fig. 3a–c). The reduced overall recovery of EGFP-Paxillin indicates that Paxillin molecules are not efficiently exchanged, suggesting that FAs in *Stonin1*^*−/−*^ MEFs contain a large proportion of immobile molecules. This is similar to *p14*^*−/−*^ MEFs, which display enlarged and hyperstable FAs due to defective endosomal delivery of factors required for FA disassembly^26^. We therefore addressed FA disassembly directly capitalizing on the fact that microtubule regrowth after Nocodazole washout induces FA disassembly^29^. Already 10 min after Nocodazole washout, WT cells had lost about 50% of their FAs, while about 90% were still retained in *Stonin1*^*−/−*^ MEFs (Fig. 3a,b). Only when WT cells had already started to generate new FAs about 30 min after washout, had the *Stonin1*^*−/−*^ cells finally lost the majority of their FAs. Collectively, these data identify Stonin1 as an important facilitator of FA disassembly.

To study whether the observed changes in FA dynamics in *Stonin1*^*−/−*^ MEFs affect FAs at steady state, we immunolabelled WT and *Stonin1*^*−/−*^ cells with Paxillin-specific antibodies. Contrary to the phenotype of *p14*^*−/−*^ MEFs^26^, we did not observe enlarged FAs, but an increased number of small adhesions with decreased Paxillin intensity (Fig. 3c–f). This suggests that altered FA dynamics in absence of Stonin1 do not only impair FA disassembly, but likely also FA maturation. In summary, Stonin1 is required for dynamic protein exchange within FAs and for their efficient disassembly.

### Loss of Stonin1 alters cell shape and motility

**Figure 4:**
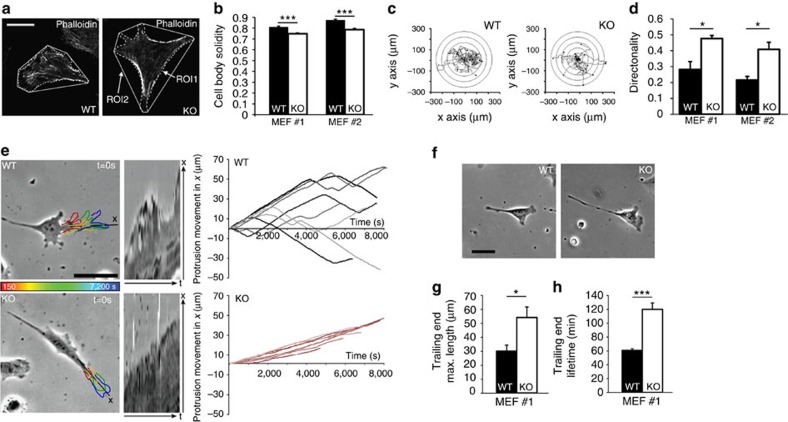
Loss of Stonin1 affects cell shape and motility. (**a,b**) Cell shape is altered in *Stonin1*^*−/−*^ MEFs. (**a**) Solidity as measure for cell shape was calculated as cell body outline (ROI1) divided by convex hull (ROI2) using fixed Phalloidin-stained WT and *Stonin1*^*−/−*^ MEFs. Scale bar, 25 μm. (**b**) Quantification of solidity in two pairs of WT and *Stonin1*^*−/−*^ MEFs (*N*=3). (**c,d**) Increased directionality of *Stonin*^*−/−*^ MEFs. Randomly migrating WT and *Stonin1*^*−/−*^ MEFs were imaged for 12–16 h, and the tracks were analysed. (**c**) Radar plots (tracks centred to their origin at t0) of WT and *Stonin1*^*−/−*^ cells. (**d**) Quantification of directionality (accumulated distance divided by Euclidean distance) for two pairs of WT and *Stonin1*^*−/−*^ MEFs (*N*=3, a minimum of two 12-h movies with a frame interval of 15 min and 30 cells were analysed per experiment). (**e**) *Stonin1*^*−/−*^ MEFs exhibit more stable protrusions. Left: representative images of WT and *Stonin1*^*−/−*^ MEFs together with kymographs along the indicated line x and the length of the movie (*t*=120 min with 150-s intervals); protrusion perimeters in 30-min intervals indicated in temporal colour code. Scale bar, 15 μm. Right: protrusion movement extracted from kymographs for WT and *Stonin1*^*−/−*^ MEFs (*n*=8). (**f**–**h**) Increased trailing end length and lifetime in *Stonin1*^*−/−*^ MEFs. Representative images (**f**) and quantification of maximum trailing end length before rear detachment (**g**) and lifetime (**h**) based on random migration movies (*N*=3, *n*=94–98). Scale bar, 25 μm. All data are depicted as mean±s.e.m. and compared by unpaired two-tailed Student's *t*-tests, ***P<0.001, *P<0.05.

FA dynamics are intimately linked to cell shape and motility. Consistent with their altered FA turnover, *Stonin1*^*−/−*^ cells exhibit an altered shape with broader and more pronounced protrusions as evidenced by their reduced solidity (cell body outline divided by convex hull) (Fig. 4a,b). To address cellular motility, we tracked the random migration of WT and *Stonin1*^*−/−*^ MEFs. WT cells frequently changed direction following a meandering path, while *Stonin1*^*−/−*^ MEFs migrated with significantly increased directionality (Fig. 4c,d), while their speed was not consistently changed (Supplementary Fig. 3d).

Directionality is influenced by the behaviour of leading edge protrusions^30^, as well as by the persistence of the trailing end^31^. Detailed observations of single cell movement revealed that WT cells extended protrusions that retracted or changed direction as the cell moved along, while protrusions of *Stonin1*^*−/−*^ cells more persistently moved forward once they were formed (Fig. 4e). Furthermore, the trailing ends of *Stonin1*^*−/−*^ MEFs had an increased length and adhered longer to the substratum before detachment (Fig. 4f–h). The enhanced directionality of *Stonin1*^*−/−*^ MEFs is, thus, presumably caused by the combined effects of greater persistence of leading edge protrusions and trailing ends.

### Stonin1 interacts with NG2 facilitating its internalization

**Figure 5:**
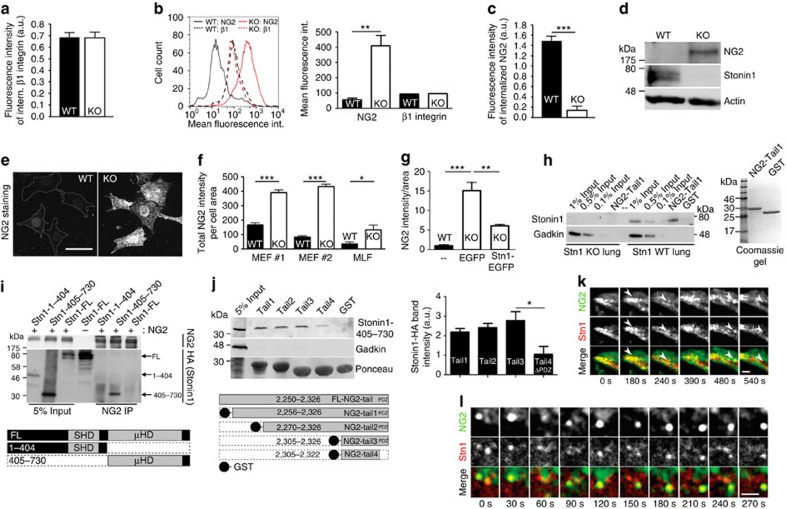
Stonin1 facilitates NG2 internalization. (**a**) Normal β1-integrin internalization in *Stonin1*^*−/−*^ MEFs. Fluorescent β1-integrin-specific antibodies internalized after 30 min were measured by flow cytometry (*N*=3). (**b**) Increased NG2 surface levels in *Stonin1*^*−/−*^ MEFs. Surface levels of NG2 and β1-integrin were analysed by flow cytometry (*N*=3). (**c**) Impaired NG2 internalization in *Stonin1*^*−/−*^ MEFs. Quantification of fluorescence intensity of internalized NG2-specific antibodies after 30 min (*N*=3). (**d**–**f**) Increased total NG2 levels in *Stonin1*^*−/−*^ MEF. (**d**) Lysates of WT and *Stonin1*^*−/−*^ cells were analysed by immunoblotting with antibodies against the indicated proteins. (**e**) Epifluorescent images of immunostained WT and *Stonin1*^*−/−*^ MEFs using NG2-specific antibodies. Scale bar, 50 μm (**f**) Quantification of total NG2 levels from images such as in **e** from two independent MEF pairs and primary MLFs (*N*=3, *n*>100). (**g**) Re-expression of Stonin1 partially rescues increased NG2 levels. Quantification of NG2 immunostainings of WT and *Stonin1*^*−/−*^ MEFs lentivirally transduced for 72 h to re-express Stonin1-EGFP or EGFP. Scale bar, 50 μm (*N*=3). (**h**) Stonin1 and NG2 form a complex. Left: bead-coupled GST-tagged NG2-cytosolic-tail resp. GST as control was incubated with lysates from WT and *Stonin1*^*−/−*^ lungs (=control). After elution from washed beads, samples were analysed by immunoblotting with Stonin1- and Gadkin-specific (=control) antibodies. Right: Coomassie gel indicating integrity of purified proteins. (**i**) The Stonin1-μHD interacts with NG2. Beads coupled to NG2-specific antibodies were incubated with lysates from HEK293T cells overexpressing full-length (FL) haemagglutinin (HA)-tagged Stonin1 or N-resp. C-terminal truncations. After elution from washed beads, samples were analysed by immunoblotting with NG2- and HA-specific antibodies. (**j**) Removal of the NG2 PDZ motif significantly impairs binding of Stonin1-μHD. Bead-coupled progressively truncated GST-tagged variants of the NG2-cytosolic-tail, as well as GST were incubated with lysates from HEK293T cells overexpressing the HA-tagged Stonin1-μHD. After elution from washed beads, samples were analysed by immunoblotting with HA- and Gadkin-specific (=control) antibodies. Right: quantification of immunoblot band intensities of HA-tagged Stonin1-405-730 (*N*=3). (**k**,**l**) Stonin1 and NG2 co-localize in living cells in protrusions (arrows point at NG2- and Stonin1-positive structures that move and disappear together) (**k**) and in mobile spots (**l**). Scale bars, 1.5 μm. Time-lapse TIRF imaging of live *Stonin1*^*−/−*^ MEFs transfected with Stonin1-tdTomato and NG2-EGFP (complete cell depicted in Supplementary Fig. 4b). All data are depicted as mean±s.e.m. and compared by unpaired two-tailed Student's *t*-tests (**b**,**c**,**f**) or one-way analysis of variance followed by Tukey's post-test (**g**,**j**), ***P<0.001, **P<0.01, *P<0.05.

The data presented so far identify Stonin1 as an important regulator of FA dynamics and cellular motility. Loss of Stonin1 impairs FA disassembly and increases migratory directionality. The similarity of Stonin1 to endocytic adaptors suggests that it controls these processes by facilitating the internalization of a specific cargo. However, so far no cargo for Stonin1 has been identified. Endocytic adaptors previously implicated in FA disassembly promote endocytosis of integrins^5^,^21^,^32^, yet, to our surprise, *Stonin1*^*−/−*^ MEFs showed normal β1-integrin uptake (Fig. 5a) suggesting that Stonin1 acts on a distinct cargo. To identify this cargo and to unravel the mechanism underlying Stonin1's impact on cell motility, we employed a proteomic approach. As the cargo protein should accumulate on the cell surface in absence of its endocytic adaptor, we determined the surface proteome of WT and *Stonin1*^*−/−*^ MEFs using stable isotope labelling with amino acids in cell culture-based quantitative mass spectrometry.

This analysis revealed a striking accumulation of the proteoglycan NG2 on the surface of *Stonin1*^*−/−*^ MEFs (fold enrichment: 34±14, *N*=2, data given as mean±s.e.m., compare Supplementary Data 1). NG2 interacts with extracellular matrix components, functions as co-receptor for PDGFR and integrins and regulates cellular motility^11^,^12^,^13^,^14^,^15^,^16^,^17^,^20^. In addition, it is a known oncogene, which has been targeted in preclinical models of glioblastoma multiforme and melanoma^33^. Fluorescence-activated cell sorting analysis of NG2 levels in WT and *Stonin1*^*−/−*^ MEFs confirmed the marked increase of NG2 at the surface of *Stonin1*^*−/−*^ cells, while β1-integrin levels were unchanged (Fig. 5b). To ascertain that elevated NG2 levels in *Stonin1*^*−/−*^ cells are caused by defective endocytosis, we performed uptake assays with NG2-specific antibodies. These demonstrated a markedly impaired internalization of NG2 in *Stonin1*^*−/−*^ cells (Fig. 5c), whereas uptake of β1-integrin proceeded unaltered (Fig. 5a).

So far endocytosis has not been described as a mechanism for the regulation of NG2, which is proposed to be removed from the cell surface by proteolysis^34^,^35^. If NG2 is endocytosed, it should be detectable in endosomal vesicles. To test this, we monitored early endosomes labelled with Rab5-EGFP, together with SNAP-tagged NG2. Indeed, coincident with its disappearance from the cell surface NG2 entered Rab5-positive endosomes (Supplementary Fig. 4a). At least a fraction of internalized NG2 is presumably degraded in lysosomes, as loss of Stonin1 did not only cause NG2 surface accumulation, but also greatly increased total NG2 levels (Fig. 5d,e). Elevated NG2 levels in absence of Stonin1 were confirmed in acutely isolated mouse lung fibroblasts (MLFs) and in a second independent WT/*Stonin1*^*−/−*^ MEF pair (Fig. 5f). In addition, we conducted rescue experiments to analyse whether the NG2 accumulation is indeed caused by Stonin1 deficiency. As expected, re-expression of EGFP-Stonin1 in *Stonin1*^*−/−*^ cells significantly reduced NG2 levels, while EGFP expression did not (Fig. 5g).

If Stonin1 acts as an endocytic adaptor for NG2, both proteins should form a transient complex. To test this hypothesis, we incubated lung lysate as an abundant source of Stonin1 with the GST-fused cytosolic tail of NG2. Indeed, the cytosolic NG2 tail was able to precipitate endogeneous full-length Stonin1 (Fig. 5h). To delineate the binding site within Stonin1, we used HEK293T cells overexpressing full-length NG2 in combination with different Stonin1 variants for co-immunoprecipitations. This revealed that NG2 binds to the Stonin1-μHD (Fig. 5i). Surprisingly, in these experiments we could not detect binding between full-length Stonin1 and NG2. This might be due to the lower expression level of ectopically expressed full-length Stonin1 as compared with the Stonin1-μHD. However, this result might also indicate that full-length Stonin1 binds less efficiently to NG2, suggesting that Stonin1 might be regulated by autoinhibition, the exact mechanism of which remains to be determined. To dissect the mode by which NG2 binds to Stonin1-μHD, we conducted affinity chromatography using progressively truncated GST fusions of the NG2 intracellular domain, which were incubated with lysates from Stonin1-μHD-expressing cells. Deleting the PDZ-binding motif QYWV at the C terminus of NG2 blocked binding of the Stonin1-μHD (Fig. 5j). We conclude from our biochemical analyses that Stonin1 associates with the C-terminal PDZ-binding motif of NG2, possibly indirectly via a PDZ domain protein, as Stonin1 lacks a PDZ domain. In addition, complex formation between NG2 and Stonin1 is also evident from their co-localization and coordinate movement in living cells (Fig. 5k,l; Supplementary Fig. 4b).

### Increased NG2 clustering in *Stonin1* ^*−/−*^ MEFs

**Figure 6:**
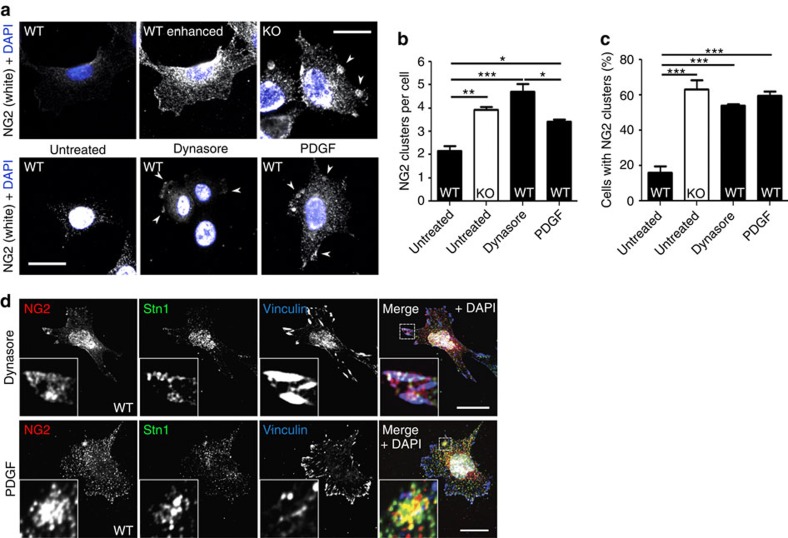
Increased NG2 clustering in *Stonin1*^*−/−*^ MEFs. (**a–c**) *Stonin1*^*−/−*^ MEFs display NG2 clusters constitutively, WT MEFs only upon stimulation or inhibition of endocytosis. (**a**) Epifluorescent images of fixed MEFs immunolabelled with NG2-specific antibodies, which were untreated or incubated with 100 μM dynasore or 50 ng ml^−1^ PDGF for 30 min. Top left and right: images of WT and *Stonin1*^*−/−*^ KO MEFs taken with same exposure; remaining WT images have enhanced contrast to visualize low levels of NG2. Arrowheads indicate clusters. Scale bar, 25 μm. Quantification of NG2 clusters per cell (**b**) and percentage of cells containing NG2 clusters (**c**) (data are depicted as mean±s.e.m., one-way analysis of variance followed by Tukey's post-test, *N*=3, *n*>182, ***P<0.001, **P<0.01, *P<0.05). (**d**) Stonin1 is present in NG2 clusters. Confocal images of WT MEFs treated with PDGF or dynasore as above and immunolabelled with antibodies against the indicated proteins. Scale bar, 25 μm. DAPI, 4,6-diamidino-2-phenylindole.

What are the consequences of impaired NG2 internalization? Clustering of NG2, for example, by antibody binding was shown to precede NG2 activation of Cdc42 (ref. 36). A comparison of the distribution of NG2 in WT and *Stonin1*^*−/−*^ cells revealed that WT MEFs contain few NG2 clusters at steady state, while these are strikingly more prominent in *Stonin1*^*−/−*^ cells (Fig. 6a–c). Inhibiting endocytosis with dynasore for 30 min increased the number of NG2 clusters also in WT cells, while NG2 levels remained unchanged on this short timescale. This indicates that the occurrence of NG2 clusters in *Stonin1*^*−/−*^ cells is not a secondary result of elevated NG2 levels, but that the clusters accumulate due to lack of NG2 internalization, confirming that NG2 clusters are normally resolved by endocytosis. Intriguingly, not only inhibition of endocytosis induces NG2 clusters in WT cells, but also stimulation with PDGF (Fig. 6a–c), suggesting that NG2 clusters serve as signalling hubs under physiological conditions. The action of PDGF on NG2 is also consistent with NG2's proposed function as a co-receptor of PDGFR, which potentiates PDGFR signalling^10^.

**Figure 7:**
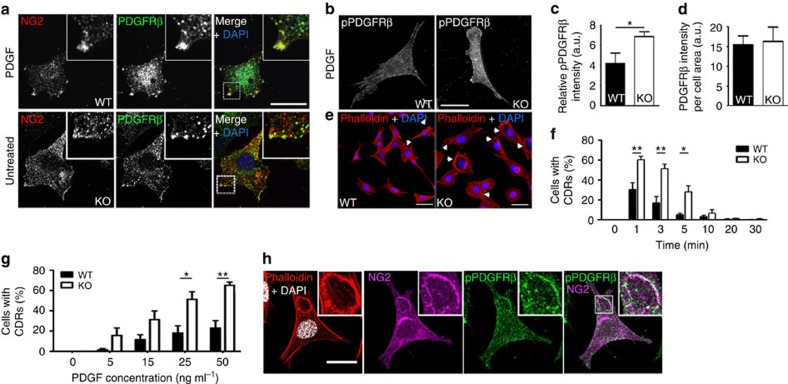
Increased PDGFR activation and circular dorsal ruffle formation in *Stonin1*^*−/−*^ MEFs. (**a**) NG2 clusters contain PDGFRβ. Confocal images of WT and *Stonin1*^*−/−*^ MEFs untreated or incubated with PDGF as in Fig. 6 and immunolabelled with antibodies against NG2 and PDGFRβ. Scale bar, 25 μm. (**b**–**d**) Increased levels of activated PDGFRβ in *Stonin1*^*−/−*^ MEFs, while total levels are unchanged. (**b**) Epifluorescent images of WT and *Stonin1*^*−/−*^ MEFs incubated with 50 ng ml^−1^ PDGF for 3 min and immunolabelled with antibodies against activated PDGFRβ (pPDGFR). Scale bar, 50 μm. (**c**) Quantification of relative fluorescence intensity of pPDGFRβ from images such as in **b** (*N*=5). (**d**) Quantification of relative fluorescence intensity of total PDGFRβ from images of WT and *Stonin1*^*−/−*^ MEFs immunolabelled with PDGFRβ-specific antibodies (*N*=3). (**e**–**f**) *Stonin1*^*−/−*^ MEFs have more CDRs upon PDGF treatment. (**e**) Example epifluorescent images of WT and *Stonin1*^*−/−*^ MEFs treated for 3 min with 50 ng ml^−1^ PDGF and immunostained with fluorescently labelled Phalloidin to visualize F-actin. Arrows point to CDRs. Scale bar, 25 μm. (**f**) Quantification of the percentage of cells with CDRs from images as in **e** after different incubation times with 50 ng ml^−1^ PDGF. (**g**) *Stonin1*^*−/−*^ MEFs need a lower PDGF concentration to trigger CDR formation. WT and *Stonin1*^*−/−*^ MEFs were treated for 3 min with the indicated concentrations of PDGF and immunostained with fluorescently labelled Phalloidin to visualize F-actin. The number of cells containing CDRs was quantified for each condition. (**h**) NG2 localizes to CDRs. Confocal image of NG2-transfected MEF incubated with 50 ng ml^−1^ PDGF for 5 min and immunolabelled with antibodies against NG2 and pPDGFRβ. Actin was visualized by fluorescently labelled phalloidin. Nuclei were stained with 4,6-diamidino-2-phenylindole (DAPI). Scale bar, 25 μm. All data are depicted as mean±s.e.m. and compared by unpaired two-tailed Student's *t*-tests, **P<0.01, *P<0.05.

If Stonin1 mediates the dissolution of NG2 clusters, it should be present in these clusters. To test this, we induced clusters in WT cells by dynasore or PDGF and performed immunostainings. Indeed, Stonin1 localizes to NG2 clusters, which are often present at protrusions and partially co-localize with FA markers (Fig. 6d). As NG2 is a known co-receptor and potentiator of PDGFR and responds to PDGF treatment, we speculated that PDGFR might likewise be present in NG2 clusters and might exhibit increased activation. In fact, NG2 clusters observed in WT cells upon PDGF treatment co-clustered PDGFR, and the receptor was also present in NG2 clusters in *Stonin1*^*−/−*^ cells (Fig. 7a). While the overall level of PDGFR remained unaltered in *Stonin1*^*−/−*^ cells, there was a significant increase in phosphorylated PDGFR (Fig. 7b–d).

### Elevated circular dorsal ruffle formation in *Stonin1* ^*−/−*^ MEFs

Activated PDGFR is known to induce circular dorsal ruffles (CDRs) in MEFs, actin-rich membrane structures that are involved in the bulk internalization of transmembrane proteins such as EGFR and in cell motility^37^,^38^. In line with enhanced activation of PDGFR in absence of Stonin1, *Stonin1*^*−/−*^ MEFs displayed elevated CDR formation as early as 1 min after PDGF stimulation (Fig. 7e,f). Moreover, *Stonin1*^*−/−*^ cells required lower doses of PDGF to initiate CDRs when compared with WT MEFs (Fig. 7g). This increased PDGF sensitivity of *Stonin1*^*−/−*^ cells is consistent with their elevated NG2 levels, as NG2 potentiates PDGFR signalling. In line with a role of NG2 in CDR formation, NG2 and activated PDGFR colocalize at CDRs (Fig. 7h).

### NG2 mediates the impact of Stonin1 on directionality

**Figure 8:**
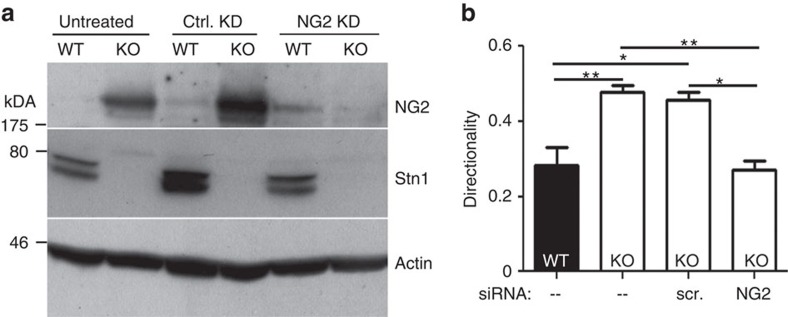
NG2 mediates the impact of Stonin1 on directionality. (**a**) Successful siRNA-mediated depletion of NG2. Lysates of WT and *Stonin1*^*−/−*^ MEF untreated or treated with scrambled control or NG2-specific siRNAs were analysed by immunoblotting with antibodies against NG2, Stonin1 and actin (KD, knockdown). (**b**) NG2 depletion rescues directionality phenotype. Quantification of directionality (accumulated distance divided by Euclidean distance) for WT and *Stonin1*^*−/−*^ MEFs, which were untreated or transfected with scrambled or NG2-specific siRNA (data are depicted as mean±s.e.m., one-way analysis of variance followed by Tukey's post-test, *N*=3, *n*>124, **P<0.01, *P<0.05).

In addition to its role as potentiator of PDGF signalling, NG2 is a known regulator of cellular motility^11^,^12^,^13^,^14^,^15^,^16^,^17^, which controls, for example, the directional migration of oligodendrocyte precursor cells^11^. Thus, the elevated NG2 levels in *Stonin1*^*−/−*^ MEFs might underlie their altered migratory behaviour. To test whether the accumulation of NG2 is causally involved in the directionality increase of *Stonin1*^*−/−*^ cells, we silenced NG2 in *Stonin1*^*−/−*^ MEFs by short interfering RNA (siRNA) and analysed their migratory pattern. Indeed, the directionality of *Stonin1*^*−/−*^ cells reverted to WT levels upon NG2 depletion, whereas a scrambled control siRNA did not influence the directionality of *Stonin1*^*−/−*^ cells (Fig. 8a,b). We thus conclude that the accumulation of NG2 causes the defective cell motility of *Stonin1*^*−/−*^ MEFs. The altered shape of *Stonin1*^*−/−*^ cells, however, was not rescued by NG2 depletion arguing for additional NG2-independent functions of Stonin1 (Supplementary Fig. 5a,b).

## Discussion

Proteoglycans such as NG2 play critical roles in cell signalling and migration and are known to promote tumourigenesis^39^. Nevertheless, their regulation is incompletely understood. We here show that NG2 function is critically controlled by endocytosis, and identify the until now uncharacterized protein Stonin1 as a dedicated adaptor for the internalization of NG2. *Stonin1*^*−/−*^ MEFs display markedly elevated NG2 levels, which are associated with altered FA dynamics and with an increase in cellular signalling and directional cell migration. The increased directional persistence of *Stonin1*^*−/−*^ MEFs is directly linked to Stonin's role as an endocytic adaptor for NG2, as NG2 depletion rescues this phenotype. Elevated NG2 levels likely augment directionality due to the interaction of NG2 with Syntenin1 (ref. 40). Cells expressing Syndecan-4 mutants with enhanced Syntenin1 binding show increased directional persistence, similar to *Stonin1*^*−/−*^ MEFs. This is caused by Syntenin-mediated suppression of Arf6 activity, which alters the integrin composition of FAs and thereby promotes directional movement^41^. Thus, elevated Syntenin recruitment due to increased NG2 levels might contribute to the increased directionality of *Stonin1*^*−/−*^ MEFs.

Cellular motility also depends on FA dynamics. Random migration requires highly dynamic FA turnover, whereas increased FA stabilization results in directionally persistent migration^42^. Our data indicate that Stonin1 is an important regulator of FA turnover. Stonin1 localizes specifically to CCPs in close vicinity of FAs, accumulates at FAs during their disassembly and facilitates the disassembly process. FAs of *Stonin1*^*−/−*^ MEFs exchange their components less efficiently and disassemble more slowly in line with the impaired removal of a crucial component. Thus, greater stability of FAs in *Stonin1*^*−/−*^ MEFs likely also contributes to increased directionality. While proteomic studies of FAs reported Stonin1 levels to increase upon the disassembly of mature FAs due to myosinII inhibition^22^,^23^, NG2 levels decreased at the same time^22^ consistent with its endocytic removal by Stonin1 during FA disassembly. Finally, persistent trailing ends of migrating cells also promote directionality^31^. Thus, the longer and more stable trailing ends of *Stonin1*^*−/−*^ MEFs, which have also been observed upon clathrin depletion^5^, likely contribute to elevated directionality as well.

While cells with FA disassembly defects such as *p14*^*−/−*^ MEFs^26^ tend to have enlarged FAs, surprisingly, we observed an increased number of small adhesions in *Stonin1*^*−/−*^ MEFs at steady state. Increased levels of small adhesions have been reported upon disorganization of the lamellar stress fibre network following depletion of Septin9 (ref. 43) or ROCK inhibition^44^. This is in line with the fact that FA maturation does not only require myosinII-dependent tension, but also actin-binding proteins that mediate the formation of the lamellar actin network^43^,^45^,^46^,^47^,^48^. The fact that *Stonin1*^*−/−*^ cells contain decreased surface-associated levels of Septin9 (fold-reduction in *Stonin1*^*−/−*^ versus WT: 0.35±0.07, *N*=2, data given as mean±s.e.m., compare Supplementary Data 1) and that Stonin1 co-immunoprecipitates actin (Fig. 1c) suggests that loss of Stonin1 might not only affect FAs by serving as an endocytic adaptor for NG2 but also by influencing the cytoskeleton, a hypothesis that awaits further testing. An NG2-independent function of Stonin1 in regards to cytoskeletal dynamics might also underlie the observed alterations in cell shape upon loss of Stonin1.

In addition to alterations in FA dynamics and cellular motility, *Stonin1*^*−/−*^ MEFs display increased PDGF-dependent signalling. This is consistent with the fact that NG2 is not only involved in cell migration but also serves as co-receptor for PDGF and potentiates PDGFR-mediated signalling^10^. Our data suggest that NG2 aggregates upon PDGF stimulation into clusters that contain PDGFR and promotes PDGFR activation. We show that these signalling clusters have to be resolved by Stonin1-mediated endocytosis of NG2 to limit PDGF signalling. By shuttling NG2 into the endosomal pathway, Stonin1 limits NG2 signalling from the plasma membrane. Failure to efficiently internalize NG2 leads to elevated signalling causing for instance an increase of PDGF-induced CDRs.

On the basis of the presented data, we propose the following model: Stonin1 resides at peripheral CCPs, which participate in the removal of FA components during FA disassembly and in the dissolution of associated signalling clusters. During these endocytic events, Stonin1 specifically binds to NG2 and facilitates its internalization. Thereby Stonin1 promotes FA disassembly and limits NG2 activity, which results in decreased persistence of movement and lower levels of cellular signalling.

In summary, we identify Stonin1 as an endocytic adaptor for NG2 and illustrate how endocytic regulation fine-tunes FA dynamics, cellular motility and signalling. As alterations in these processes are intimately linked to tumorigenesis and as NG2 is a well-known oncogene, Stonin1 might be crucial for limiting tumour growth by keeping NG2's oncogenic potential in check via its removal from the plasma membrane. Future studies involving tumour models will have to validate the putative tumour suppressor function of Stonin1.

## Methods

### Reagents

For information on antibodies, and plasmids and siRNAs see Supplementary Tables 1 and 2.

### Generation and genotyping of *Stonin1* ^*−/−*^ mice

The mouse Stonin1 gene (*Ston1*) is located on chromosome 17 and consists of three coding exons according to Ensembl entry ENSMUST00000064035. *Stonin1*^*−/−*^ mice were generated by replacing the first coding exon, which comprises most of the protein-coding sequence, by a floxed neomycin-resistance cassette using homologous recombination in embryonic stem cells (Supplementary Fig. 1), which were injected into C57BL/6 blastocysts and subsequently implanted into adult pseudopregnant C57BL/6 mice. The resulting chimeric males were mated with Ella-Cre Deleter mice to remove the neomycin-resistance cassette. The obtained Stonin1 mice with one disrupted *Ston1* allele did not show any overt phenotypic differences to WT animals. They were backcrossed onto a C57BL/6 J background and interbred to obtain WT and *Stonin1*^*−/−*^ littermates for experiments (line name: Ston1^tm1.1Tmar^). *Stonin1*^*−/−*^ mice did not show any overt phenotypical differences. Animals were genotyped before experiments by PCR analysis of genomic DNA obtained from tail or ear biopsies. The *Ston1* WT allele was detected with the forward primer TM52 (5′- CTGGACCAGGAACCTTCAGA -3′) and the reverse primer TM95 (5′- CGAGCAAGCACTCATCTCTC -3′) (product: 402 nt). The *Ston1* KO allele was detected using the forward primer TM95 and the reverse primer TM96 (5′- GAGTGTAGAGTGTGGCGCTC -3′) (product: 301 nt). Mice were housed in small groups in standard cages and kept on a 12-h dark–light cycle with food and water *ad libitum*. All animal experiments in the present study were conducted in strict accordance with ethical regulations and the animal welfare guidelines of the Landesamt für Gesundheit und Soziales (LAGeSo) Berlin and with their permission.

### Generation of MEFs

E13.5 embryos of random sex were transferred into sterile ice-cold Hanks' balanced salt solution (HBSS). After removal of head and inner organs (which were kept for genotyping), embryonic tissue was dissected into small parts and digested in 5 ml trypsin/EDTA for 15 min at 37 °C in a shaking water bath. After brief trituration of the tissue segments, 10 ml culture medium (DMEM containing 4.5 g l^−1^ glucose, 10% FCS, 100 U ml^−1^ penicillin and 0.1 mg ml^−1^ streptomycin) were added, and cells were centrifuged at 400*g* for 5 min at room temperature, washed once with HBSS and plated in culture medium on one 10-cm culture dish per embryo and grown at 37 °C and 5% CO_2_. After 1 day *in vitro* (DIV), cell cultures were washed twice with sterile PBS, and fresh culture medium was added. Cells were passaged once before immortalization by Lipofectamin 2000 (Invitrogen)-mediated transfection with SV40 large T-antigen on DIV 10. Two independent MEF cell line pairs from WT and *Stonin1*^*−/−*^ embryos were established (pair #1 and #2).

### Generation of primary MLFs

Lungs were dissected from 5–7-day-old sex-matched Stonin1 WT and KO mice (C57BL/6J) of both genders and transferred into ice-cold HBSS. Bronchi and connective tissue were removed, and the remaining tissue was dissected into small pieces, washed three times with ice-cold HBSS and digested with 37 °C warm 20 mg ml^−1^ collagenase (Millipore) in DMEM containing 10 mg ml^−1^ bovine serum albumin (BSA) for 45 min at 37 °C in a shaking water bath. Digestion was stopped by the addition of culture medium, and tissue pieces were washed once in culture medium. After tissue trituration, cells were washed with culture medium, plated onto one 10-cm culture dish in fresh culture medium and grown at 37 °C and 5% CO_2_. The cultures were washed with PBS on DIV 1 and passaged 1:10 every 5 days.

### Cell culture and transfections

Primary cells (MEFs and MLFs) as well as HEK293T cells (obtained from ATCC) were grown in culture medium at 37 °C and 5% CO_2_. While HEK293T cells were transfected with plasmid DNA by calcium phosphate transfection according to standard protocols, MEFs were transfected either by electroporation with the Amaxa Nucleofector IIb (Lonza) using the program A-23 designated for MEFs or by lentivirus-based transduction. For lentivirus production, the lentiviral packaging plasmid psPax2 and the VSV-G envelope-expressing plasmid pMD2.G were transfected together with a Stonin1-encoding pRRLSIN.cPPT.PGK-GFP.WPRE-based plasmid into HEK293T cells. After 20 h, the transfection medium was removed and fresh culture medium was added. At 48 h post transfection, the medium was collected, debris was removed by centrifugation for 10 min at 6,000*g* and the supernatant was filtered through a 0.45-μm filter before immediate use. For lentivirus-based transductions, MEFs were incubated at 60% confluency with fresh lentivirus supernatant. After 20 h, supernatants were removed, and fresh culture medium was added. Transfection of siRNAs into MEFs was performed with RNAiMax (Invitrogen) according to the manufacturer's instruction. On day 2, the transfection medium was exchanged for fresh culture medium. At 36 h after the medium exchange, a second round of siRNA transfection was performed for 4–6 h or overnight. Directly after removal of the transfection medium, cells were split according to the planned application. Experiments were done on days 4–7. When seeding transfected cells on glass surfaces for microscopy, these surfaces were first coated with Matrigel.

### Preparation of lysates and immunoblot-based analysis

Cultured cells were washed briefly with PBS and scraped into lysis buffer (20 mM HEPES pH 7.4, 100 mM KCl, 2 mM MgCl_2_, 2 mM PMSF, 1% Triton X-100, 0.6% protease inhibitor cocktail (Sigma)). After 5 min on ice, lysates were centrifuged for 5 min with 17,000*g* at 4 °C. The protein concentration of the supernatant was determined by the Bradford assay. Tissue extracts were obtained by tissue homogenization in homogenization buffer (320 mM sucrose, 4 mM HEPES pH 7.4, 2 mM PMSF, 0.3% protease inhibitor cocktail (Sigma)) using a potter (15 strokes at 1,000 r.p.m.). After centrifugation with 1,000 g at 4 °C for 10 min, the supernatant was collected and again cleared by centrifugation for 5 min with 17,000*g* at 4 °C to yield the final tissue extract. Tissue lysates used for binding experiments were adjusted to 1 × lysis buffer and lysed for 10 min on ice before centrifugation for 15 min with 43,500*g* at 4 °C and again for 15 min with 265,000*g* at 4 °C in an ultracentrifuge to obtain a clear lysate before determining the protein concentration by the Bradford assay. Before immunoblotting lysates were adjusted to 1 × Laemmli sample buffer, samples were analysed by sodium dodecyl sulfate polyacrylamide gel electrophoresis (SDS-PAGE) and immunoblotting. Bound primary antibodies were detected by incubation with secondary antibodies conjugated to horseradish peroxidase (Jackson ImmunoResearch; dilution: 1:10,000) and a chemiluminescent substrate. Uncropped versions of all immunoblots are depicted in Supplementary Fig. 6.

### MEF treatments

To assess effects of cellular stimulation, cells were either left untreated or washed twice in sterile PBS and starved 12–48 h in serum-free DMEM. Cells were then stimulated with either 10% BCS or with 50-100 ng ml^−1^ PDGF-BB (Peprotech (termed PDGF in the text)), if not stated otherwise in the figure legend. Cultures were stimulated from 1 to 60 min before immediate lysis on the dish as described above. The dynamin inhibitor Dynasore (self-synthesized) was applied at 100 μM for 30 min. The myosinII inhibitor blebbistatin (Merck-Millipore) was applied at 25 μM for 5–30 min.

### Surface biotinylation and affinity purification

MEFs were cultured for 6 days in L-lysine- and L-arginine-free DMEM/10% FCS (Thermo Fisher Scientific Inc.) supplemented either with the ‘heavy' amino acids ^13^C_6_-L-lysine and ^13^C_6_,^15^N_4_-L-arginine (Silantes GmbH) in case of KO MEFs or normal ‘light' amino acids in case of WT MEFs. For biotinylation of the surface, protein pool cells were placed on ice, washed twice with ice-cold PBS and incubated with 0.5 mg ml^−1^ Sulfo-NHS-LC-Biotin (EZ-Link, Pierce/Thermo Scientific) in PBS while shaking for 30 min at 4 °C. The biotinylation solution was removed and surplus biotin was quenched by two 5-min washes with 50 mM glycine in PBS at 4 °C on a shaker. Cells were harvested, and lysates were prepared as described above. After protein determination by the Bradford assay, WT and KO lysates were brought to the same concentration and mixed 1:1. Biotinylated molecules were isolated by a 1-h incubation of lysates with streptavidin beads on a rotator at 4 °C. After centrifugation at 3,500*g*, the supernatant was transferred to a fresh tube. Beads were washed extensively and bound protein was eluted with Laemmli buffer and separated by SDS–PAGE.

### Relative protein quantification by mass spectrometry

For liquid chromatography (LC)–mass spectrometry (MS)/MS analysis, coomassie-stained lanes were cut into slices and proteins were digested with trypsin as described^49^. In brief, gel slices were washed with 50% (v/v) acetonitrile in 50 mM ammonium bicarbonate, dehydrated in acetonitrile and dried in a vacuum centrifuge. The dried gel pieces were reswollen in 15 μl of 50 mM ammonium bicarbonate containing 60 ng trypsin (sequencing grade, Promega). After 17 h incubation at 37 °C, 15 μl of 0.3% trifluoroacetic acid in acetonitrile were added, and the separated supernatant was dried under vacuum and redissolved in 6 μl of 0.1% trifluoroacetic acid, 5% acetonitrile in water. Tryptic peptides were analysed by a reversed-phase capillary LC system (Eksigent NanoLC Ultra, Axel Semrau GmbH, Germany) connected to an LTQ-Orbitrap XL mass spectrometer (Thermo Scientific). LC separations were performed on a capillary column (PepMap100, C18, 3 μm, 100 Å, 250 × 75 μm inner diameter, Dionex, Idstein, Germany) at an eluent flow rate of 250 nl min^−1^ using a linear gradient of 3–50% B in 65 min. Mobile phase A contained 0.1% formic acid (v/v) in water, mobile phase B contained 0.1% formic acid in acetonitrile. Mass spectra were acquired in a data-dependent mode with one MS survey scan (with a resolution of 30,000) in the Orbitrap and MS/MS scans of the four most intense precursor ions in the LTQ. Identification and quantification of proteins were carried out with version 1.2.0.18 of the MaxQuant software package as described^50^. Data were searched against an international protein index (IPI) mouse protein database (version 3.68). The mass tolerance of precursor and sequence ions was set to 20 p.p.m. and 0.35 Da, respectively. Methionine oxidation and the acrylamide modification of cysteine were used as variable modifications. False discovery rates were <1% based on matches to reversed sequences in the concatenated target-decoy database. Proteins were considered if at least two sequenced peptides were identified. The experiments were done in biological replica (two independent stable MEF lines) with reverse isotope labels. Proteins were sorted by isotope ratios. A protein can be considered as relevant if the heavy/light (experiment A) or light/heavy (experiment B) isotope ratio is higher than two in both experiments (Supplementary Data 1).

### Co-immunoprecipitations

For immunoblot analysis, the antibodies were coupled reversibly to A/G sepharose. Lysates prepared as detailed above were incubated with the antibody-coupled beads for 1–4 h at 4 °C on a rotator. Next, beads were washed extensively. Protein was eluted in Laemmli sample buffer by heating to 95 °C for 5 min, or 65 °C for 15 min in case large transmembrane proteins were to be detected. Samples were separated by SDS–PAGE and analysed by immunoblotting.

### Affinity chromatography

GST fusion proteins were expressed in *Escherichia coli* and purified from benzonase-treated bacterial lysates (to remove possible nucleic acid contaminants) using GST-bind resin (Novagen) according to standard protocols. Protein extracts from transfected cells were prepared as detailed above for incubation with GST-AP-2-α-ear, however, in lysis buffer containing 50 mM Tris pH 8, 150 mM NaCl, 1% IGEPAL and 0.6% protease inhibitor cocktail (Sigma) in case of incubation with GST-NG2-tails. For affinity purifications, GST fusion proteins were incubated with lysates for 2 h at 4 °C. Following extensive washes, bound proteins were eluted in Laemmli sample buffer. Samples were analysed by SDS–PAGE and immunoblotting.

### Immunofluorescence and live-cell imaging

For immunofluorescent stainings, cells grown on glass coverslips (CSs) were either washed with PBS and fixed with 4% PFA in PBS for 10 min at room temperature or, in the case of stainings with Stonin1-specific antibodies, they were transferred onto ice, washed with ice-cold PBS and fixed immediately with ice-cold 4% PFA in PBS for 7 min. Fixed cells were washed twice with PBS, blocked and permeabilized with goat serum dilution buffer (GSDB: 10% goat serum in PBS) containing 0.3% Triton X-100 for 30 min at room temperature for staining intracellular epitopes. For surface stainings, GSDB without detergent was used. Primary antibodies were applied in GSDB for 1–2 h at room temperature, then CSs were washed 3 × shortly in PBS. When staining pPDGFRβ, fixed cells were permeabilized for 5 min with 0.3% Triton X-100 in PBS, washed 1 × with PBS, blocked with 5% BSA in PBS for 1 h and incubated with primary antibodies overnight at 4 °C. In all cases species-specific secondary antibodies fluorescently labelled with Alexa dyes 488, 568 or 647 (Invitrogen) were applied at 1:200 in GSDB for 1 h at room temperature in the dark. To visualize F-actin, Alexa568-coupled Phalloidin (Invitrogen) was applied at a 1:50 dilution in GSDB along with the secondary antibodies. After the incubation, CSs were washed three times with PBS and mounted onto microscopy slides with ImmuMount mounting solution (Thermo Electron) supplemented with 1 μg ml^−1^ 4,6-diamidino-2-phenylindole (DAPI) to stain nuclei. For fluorescence intensity quantifications immunofluorescent images were acquired on a Nikon Eclipse Ti epifluorescent microscope, equipped with an Andor sCMOS camera, an Okolab incubator for life cell imaging and a Nikon PerfectFocus autofocus system. The set-up was operated by Micromanager 1.4.14 (ref. 51). For protein localization in *z* or protein co-localization either spinning disc confocal microscopy was employed using a Zeiss Axiovert 200M microscope (Carl Zeiss, Jena, Germany) operated by the software Volocity, equipped with an EM-CCD-camera and featuring a View ERS Rapid Confocal Imager (PerkinElmer, London, England) or for fixed specimen laser scanning microscopy using ZeissLaser Scanning Microscopes LSM510 and LSM710. Images were exported from Zen 2012 software and processed in ImageJ. TIRF was performed on the Nikon set-up described above extended with a × 60 TIRF objective and a custom-built solid-state laser set-up. Time-lapse movies of living cells were taken over time spans of 30–60 min with 20–30-s intervals between the images to follow cellular movement and protein motility inside protrusions. To minimize exposure to the lasers, pixels were binned 2 × 2 from 1,776 × 1,760 pixels. Transfected cells were imaged on Matrigel-coated 24 mm CSs in live-cell imaging solution (HBSS containing Ca^2+^, Mg^2+^, 5% FCS, 20 mM HEPES pH 7.4) at 37 °C without CO_2_. NG2 was engineered to contain an extracellularly located SNAP tag. SNAP surface Alexa647 (New England Biolabs) was used at 1:200–1:500 as SNAP substrate, a non-membrane-permeable probe that can be used to specifically label extracellular SNAP tags.

### Fluorescence recovery after photobleaching

For FRAP experiments, Paxillin-GFP transfected MEFs on Matrigel-coated CSs were transferred to imaging solution. Several protrusive FAs per cell as well as a background region were selected for photobleaching (bleaching field 15 × 15 μm; 500 ms; 488 nm) and monitored for recovery over 60 s at 1 s per frame on the spinning disc confocal microscope. FRAP data together with pre-bleach images were exported from Volocity software and further analysed in ImageJ. Fluorescence intensity plots of each FA were normalized to bleaching before averaging per cell and corrected for background, enabling comparisons between cells. The resulting data from each experiment was fitted with a single exponential recovery curve using the software Origin to calculate the half-time of recovery (*t*_1/2_) and the mobile fraction. Only experimental data for which the fit resulted in an *R*^2^–value >0.95 was included in the analysis.

### NG2 antibody uptake assay

Endocytosis of NG2 was assessed by quantification of internalized mouse-α-NG2.EC antibody against the extracellular domain of NG2. MEF cells were incubated on ice at 4 °C for 1 h with NG2.EC diluted 1:100 in uptake solution (3% BSA in DMEM). The acid quench control sample was incubated for 90 s in acid quench buffer (100 mM Na acetate, 100 mM NaCl, pH 5.3) to wash off surface-bound antibody. The acid quench control and the sample for surface pool labelling were fixed in 4% PFA in PBS for 7 min on ice. The antibody uptake was performed in the continued presence of NG2.EC in uptake solution by shifting the remaining samples from 4 to 37 °C. Samples were removed at various time points, acid quenched and fixed. After completion of all uptakes, the samples were blocked and permeabilized, incubated with an Alexa488 goat α-rabbit secondary antibody and mounted as described above. Values obtained for internalized NG2 were normalized to surface amount and subtracted for acid wash background.

### Flow cytometry

Flow cytometry was used to quantify surface levels of NG2 and β1-integrin and to perform an integrin uptake assay. For surface stainings, cells were removed from dishes by treatment with 100 mM EDTA in PBS for 5 min at 37 °C. EDTA was diluted by the addition of PBS, and cells were centrifuged at 4 °C for 5 min at 300*g*. The pellet was resuspended in PBS, and cells were fixed for 30 min on ice by adding PFA to a final concentration of 2%, and then washed once in PBS. The antibodies were applied in 3% BSA in PBS for 1 h at room temperature. Surface NG2 was stained with NG2.EC (1:100), surface integrin with a phycoerythrin (PE)-conjugated β1-integrin-specific antibody (1:50). Cells were washed once with PBS. In the case of the NG2 staining, fixed cells were incubated with a secondary mouse-anti-rabbit antibody conjugated to Alexa488 diluted 1:200 in 3% BSA in PBS for 30 min at room temperature in the dark. Finally, cells were washed with PBS and dispersed by vortexing in PBS before the flow cytometric measurement. Samples were measured with a FACSCalibur (BD Biosciences) and CellQuest Pro and analysed with FlowJo V10.

The β1-integrin internalization assay was done in essence as described for the NG2 antibody uptake assay. Briefly, MEFs attached to CSs were surface-stained with a PE-conjugated β1-integrin-specific antibody (1:50) at 4 °C for 1 h. Acid treatment was done as described for the NG2 antibody uptake. Cells were detached with 100 mM EDTA in PBS for 5 min at 37 °C, fixed and subjected to flow cytometric analysis as described above. Values obtained for internalized β1-integrin were normalized to surface amount and subtracted for acid wash background.

### FA disassembly assay

MEFs seeded on glass CSs were serum starved for 24 h. Next, they were treated for 4–5 h with 10 μM nocodazole to completely depolymerize microtubules. During washout, nocodazole was replaced with serum-free medium for the indicated time intervals to allow microtubule regrowth. Cells were then fixed, processed for immunofluorescence as described above and imaged on an epifluorescence microscope. FA numbers were quantified by particle analysis with customized ImageJ macros (for details see Data analyses). Mean values were normalized to the mean of the WT control before relative FA disassembly per genotype was calculated.

### Migration assays

Time-lapse movies of randomly migrating cells were acquired on the Nikon microscope specified above with a × 10 objective and a plate incubation chamber at 37 °C and 5% CO_2_ for 12–16 h with 15-min intervals. Cells were tracked manually with the ImageJ macro MTrackJ, and tracks were analysed with the free Chemotaxis and Migration Tool from Ibidi (Munich, Germany). Accumulated distance along the track and Euclidian distance were measured in μm, velocity was calculated as the accumulated distance divided by the time and directionality was calculated as the ratio of Euclidian distance to accumulated distance. To assess protrusion dynamics, cell migration was monitored on the same set-up in 150-s intervals for 120 min. Protrusion dynamics were analysed using the resource function of ImageJ.

### Data analyses

Image analysis was performed with Fiji, an ImageJ 1.47g package^52^. Processing was semi-automated by the use of custom-made macros in ImageJ using intensity quantifications on manually outlined cells or automatically selected particles after application of a common threshold. For three-dimensional displays, image stacks were converted using the implemented three-dimensional Project function. Time-lapse movies were created from single Tiff images. Stonin1 channels from TIRF movies were always corrected for bleaching with a specified plugin (BleachCorrectStackNew.js). This plugin normalizes the fitted exponential decay curve of a bleached sample determined from a region of interest (ROI) (bleach) to a linear function for all pixels of the movie stack. Its application results in clearer signals over background, but also increases the extracellular background artificially. To objectively evaluate the distribution of fluorescent signals in a cell, the previously described Clock Scan tool^53^ was adapted into a Java ImageJ plugin. The tool performs line scans along the radius of an amorphous shape from the centre of mass towards the selected perimeter (ROI) for all angles and normalizes the respective radius from 0 (centre) to 1 (ROI). Hence, the result is a normalized profile from the centre outwards. An advanced mode permits the selection of specified angles to scan, which allows the division of a migrating cell in front and rear. Signal intensities can be normalized, and the distance from 0 to 1 can be binned according to the user's needs to compare the profile of different shapes. Co-localization of pixels between one channel and another and vice versa was judged by quantifying Mander's coefficients.

### Statistical analyses

Values are always depicted as mean±s.e.m. Statistical significance of data was analysed by two-tailed unpaired Student's *t*-tests in case of two experimental groups, or one-way analysis of variance followed by either a Tukey or a Dunnett post-test in case of >2 experimental groups using GraphPad Prism software, and are indicated in the following way: ****P<0.0001; ***P<0.001; **P<0.01; *P<0.05. Data with arbitrary values, such as fluorescent intensities, was normalized to the mean of all samples before performing statistics. Fold increases were calculated as normalization to the respective reference, which mostly is the untreated WT sample. The number of experimental replica or animals used is given as ‘*N*', the number of analysed cells is given as ‘*n*'. No statistical method was used to predetermine sample size. If samples were excluded, the exclusion criteria are described in the respective method section. Whenever possible, data were evaluated in a blinded manner.

## Additional information

**How to cite this article**: Feutlinske, F. *et al*. Stonin1 mediates endocytosis of the proteoglycan NG2 and regulates focal adhesion dynamics and cell motility. *Nat. Commun.* 6:8535 doi: 10.1038/ncomms9535 (2015).

## Supplementary Material

Supplementary Information Supplementary Figures 1-6, Supplementary Tables 1-2 and Supplementary References

Supplementary Movie 1 Accumulation of Stonin1 upon focal adhesion disassembly. Life-cell TIRF time-lapse movie of Stonin1-/- MEF transfected with Stonin1-tdTomato and Paxillin-GFP (see Fig. 2c-f).

Supplementary Data 1 SILAC-based mass spectrometric analysis of the surface proteome of WT and Stonin1-/- MEFs.
