# Supplementary material for: Stonin1 mediates endocytosis of the proteoglycan NG2 and regulates focal adhesion dynamics and cell motility
Source: Nat Commun. 2015 Oct 5;6:8535. doi: 10.1038/ncomms9535 (PMC4600748; doi:10.1038/ncomms9535)
Supplement: Supplementary Information — Supplementary Figures 1-6, Supplementary Tables 1-2 and Supplementary References [file ncomms9535-s1.pdf]

SUPPLEMENTARY INFORMATION

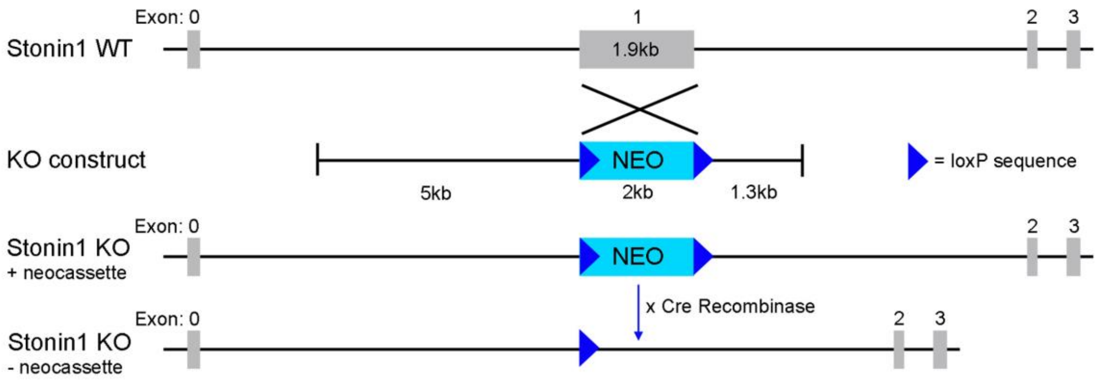

Supplementary Figure 1
Targeting strategy for the generation of *Stonin1*$^{-/-}$ mice. Scheme of the targeting strategy used for the generation of *Stonin1*$^{-/-}$ mice. The first coding exon (exon 1), which comprises most of the protein coding sequence, was replaced by a floxed neomycin resistance cassette using homologous recombination in embryonic stem cells. Subsequently the neomycin resistance cassette was removed via interbreeding with Ella-Cre Deleter mice leaving behind a single loxP sequence between the non coding exon 0 and exon 2.

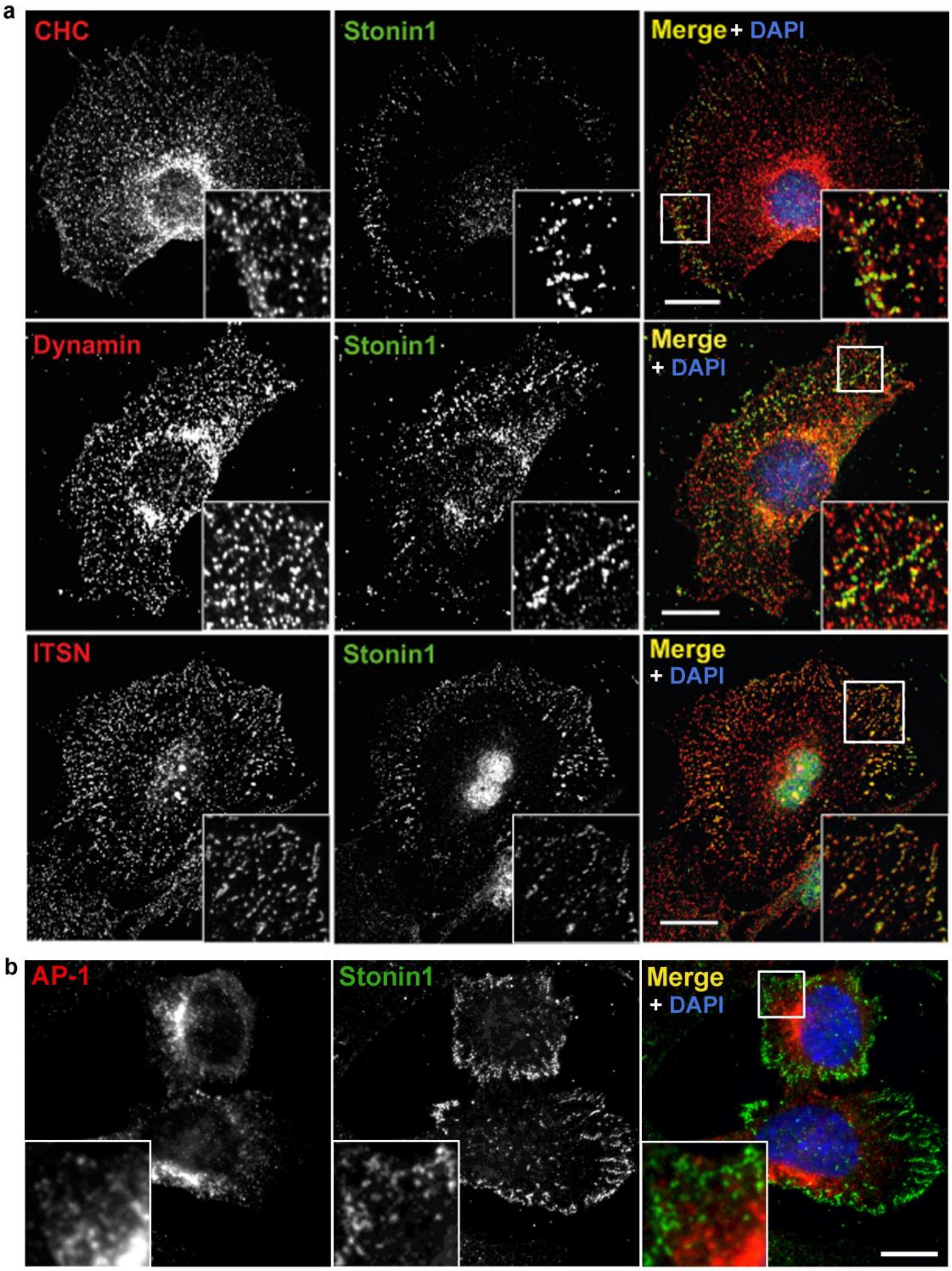

Supplementary Figure 2

Co-localization of Stonin1 with endocytic markers. (**a,b**) Stonin1 co-localizes with the endocytic proteins Clathrin (CHC), Dynamin and Intersectin (ITSN) (**a**), but not with the predominantly perinuclearly localized endosomal adaptor complex AP-1 (**b**). Fixed WT and *Stonin1*$^{-/-}$ MEFs were immunolabelled with antibodies specific for the indicated proteins and analysed by confocal microscopy. Insets show enlargements of boxed areas. Scale bar: 25 µm.

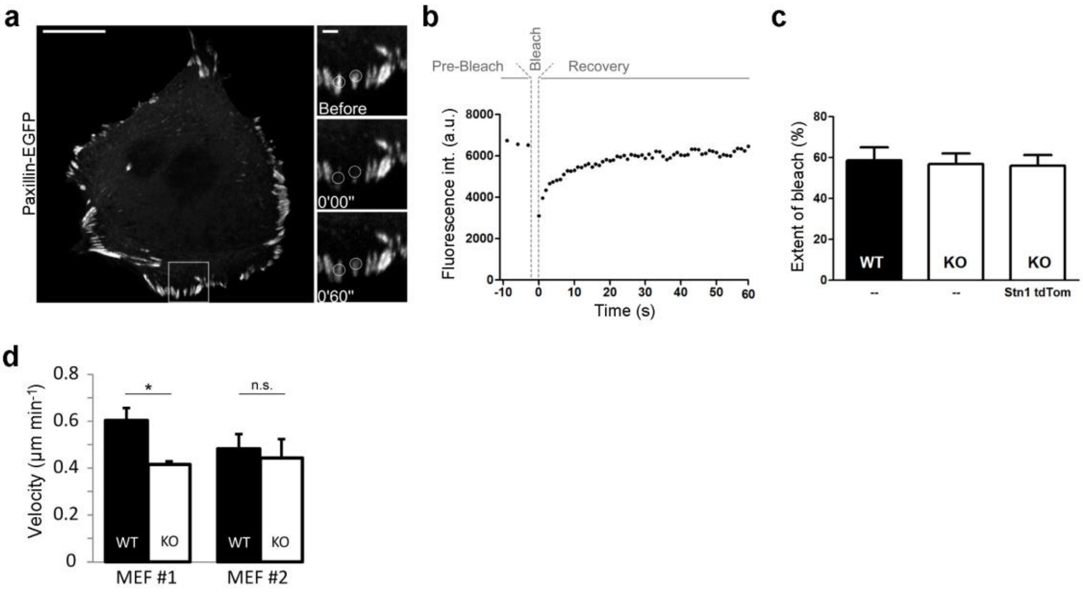

Supplementary Figure 3

FA dynamics and velocity of cell migration in absence of Stonin1. (**a-c**) FRAP of Paxillin-EGFP transfected MEFs. **(a)** Representative image of a Paxillin-EGFP transfected MEF. The boxed area shows FAs before bleaching, immediately after bleaching and 60 s after bleaching. Scale bar large image: 20 µm; scale bar inset: 5 µm. **(b)** Example for the Paxillin-EGFP fluorescence intensities measured before bleaching (Pre-bleach) and during 60 s of recovery. **(c)** Quantification of the extent of bleaching of Paxillin-EGFP fluorescence induced by the bleach pulse for the experiments depicted in Fig. 2g-h (data are depicted as mean±SEM, N=4). (**d**) No consistent change in the velocity in *Stonin1*$^{-/-}$ MEFs. Randomly migrating WT and *Stonin1*$^{-/-}$ MEFs were imaged for 12-16 h, and the tracks were used to quantify migration velocity for two pairs of WT and *Stonin1*$^{-/-}$ MEFs (data are depicted as mean±SEM, N=3, a minimum of two 12 h movies with a frame interval of 15 min and 30 cells were analysed per experiment, unpaired two-tailed Student´s t-test, *=$p<0.05$).

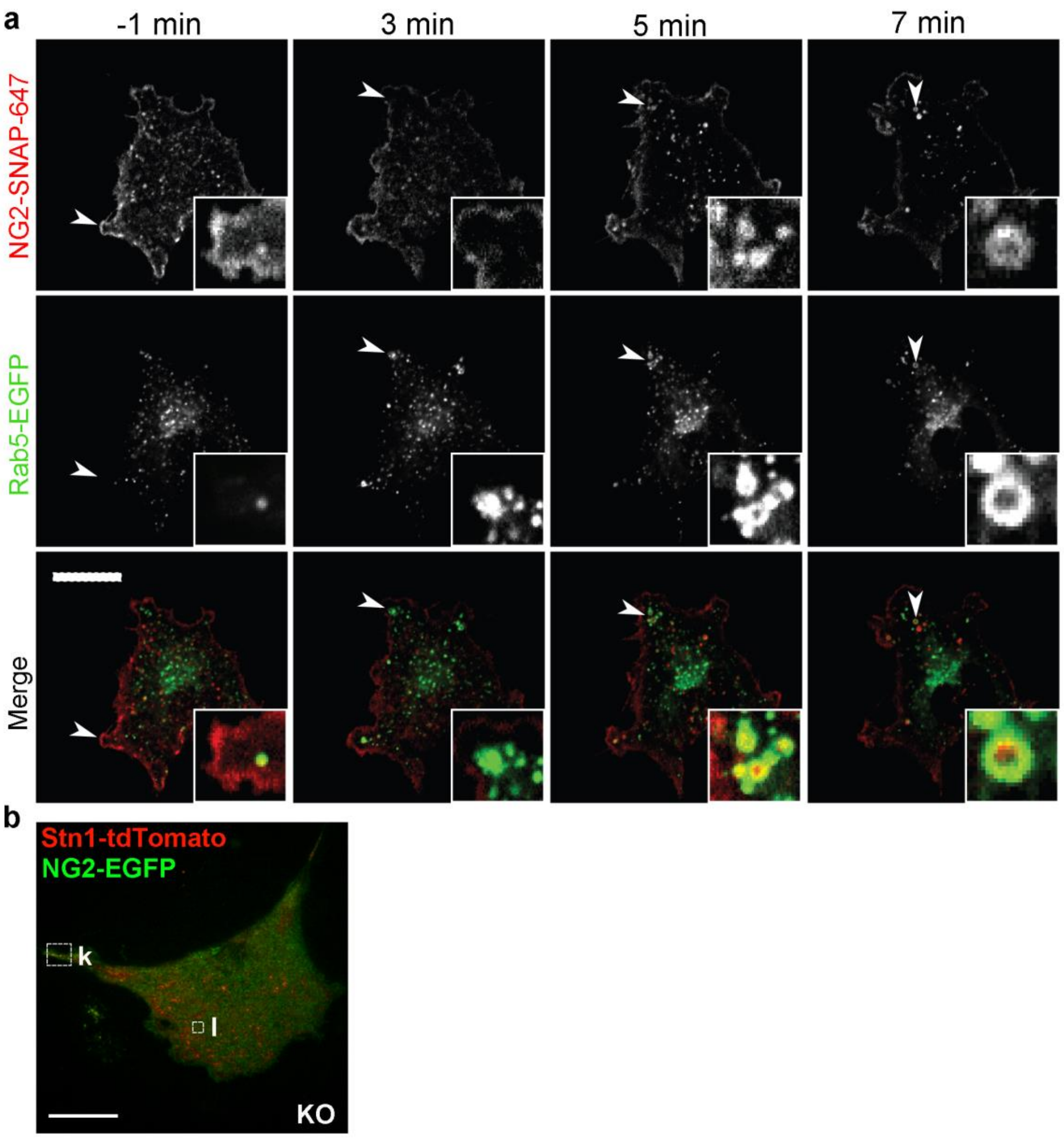

Supplementary Figure 4

NG2 trafficking. (**a**) NG2 traffics via Rab5-positive early endosomes. WT MEFs were transfected with Rab5-EGFP and NG2-SNAP, starved overnight and labelled with Alexa647-surface-SNAP-substrate for 30 min on ice before imaging for 40 min at 30 s intervals in HBSS. After 2 min 50 ng ml^{-1} PDGF were applied to the cells. The insets show the areas highlighted by the respective arrow heads. Scale bar: 25 µm. (**b**) Complete image of the Stonin1-tdTomato and NG2-EGFP transfected *Stonin1*$^{-/-}$ cell imaged by life-cell TIRF (40 min time-lapse image series with 30 s intervals) on which Figures 5k-l (corresponding to boxed areas) are based. Scale bar: 15 µm.

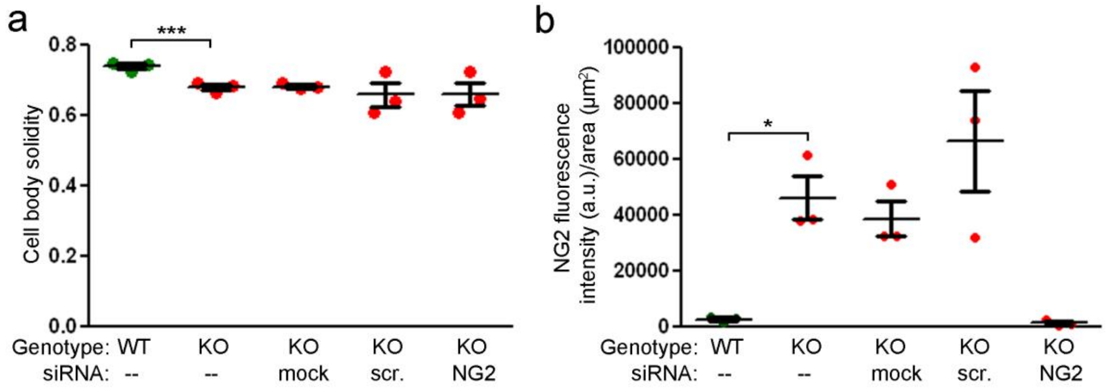

Supplementary Figure 5

The alterations in cellular shape upon loss of Stonin1 are independent of NG2. (**a**) Quantification of solidity in WT and *Stonin1*$^{-/-}$ MEFs. *Stonin1*$^{-/-}$ cells were treated with NG2-specific siRNA to deplete NG2 or with scrambled (scr.) or no siRNA (mock) as controls for the knockdown procedure (data are depicted as mean±SEM, N=3, paired two-tailed Student´s t-test, ***=p<0.001). (**b**) Quantification of NG2 fluorescence intensity in the differently treated cells to confirm the siRNA mediated depletion of NG2 (data are depicted as mean±SEM, N=3, paired two-tailed Student´s t-test, *=p<0.05).

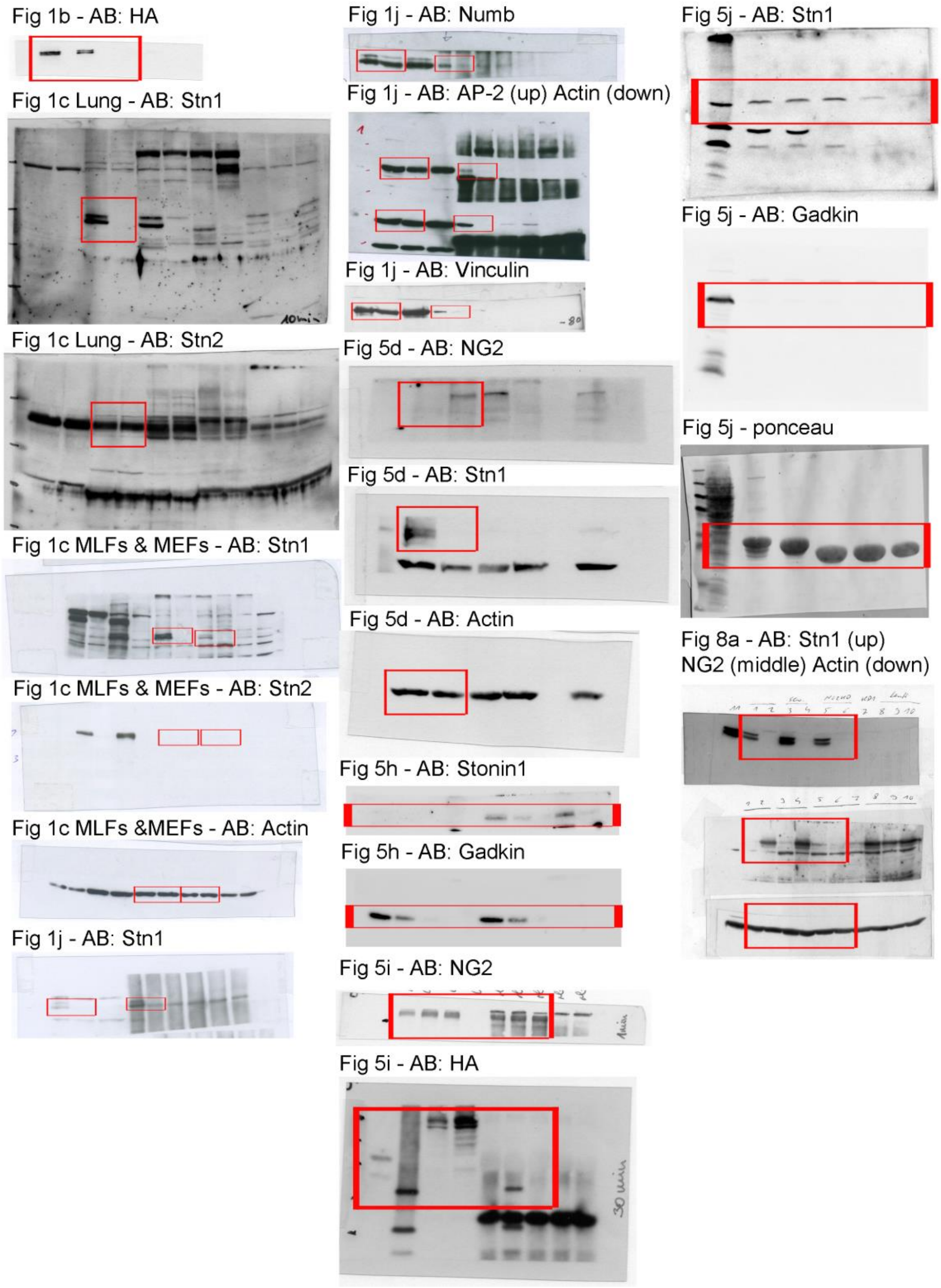

Supplementary Figure 6
Uncropped western blot images for Figures 1, 5 and 8.

Supplementary Table 1 Antibodies used in the study

Antibody specific for	spe-cies	clone name /catalog #	supplier	IF	WB	Comments
Actin	ms	ac-15 / A5441	Sigma-Aldrich		1:1000	ascites, concentration not given by supplier
AP-1	ms	610386	BD Biosciences	2.5 µg ml^{-1}		specific for γ-adaptin
AP-2	ms	AP6	Hybridoma bank	1:100	1:100	self-purified, concentration not known
CHC	ms	X22	Hybridoma bank	1:50		self-purified, concentration not known
Dynamin2	ms	27 / 610264	BD Bioscience	50 µg ml^{-1}	0.25 µg ml^{-1}	
Gadkin	rb	#13	home-made		2 µg ml^{-1}	purified[1]
HA	ms	HA.11/16 B12 MMS-101R	Babco/Covance		2-3 µg ml^{-1}	ascites
β1-integrin	ha	CD29 / eBioHMb1-1 / 12-0291	eBioscience			PE-conjugated, extracellular epitope (used 1:50 for FACS)
Intersectin	ms	ITSN (29) / sc-136242	Santa Cruz	1 µg ml^{-1}		
NG2	rb	NG2.K1	J. Trotter			12 µg used per IP
NG2	rb	NG2.553	W. Stallcup	1:500		antibody mixture against rat NG2, concentration not known
NG2	gp	NG2gp	W. Stallcup	1:20		specific to rat NG2 extracellular domain aa 30-2225, concentration not known
NG2	ms	NG2.EC	W. Stallcup	1:100		specific to extracellular domain of rat NG2, concentration not known
NG2	ms	MAB5384	Chemicon/ Millipore	10 µg ml^{-1}	2 µg ml^{-1}	recognizes only rat NG2 (used for overexpressed rat NG2)
Numb	gt	ab4147	Abcam	5 µg ml^{-1}		
Paxillin	ms	177/Paxillin / 610569	BD Transduction	2.5 µg ml^{-1}		
PDGFRβ	rb	958 / sc-432	Santa Cruz	2 µg ml^{-1}		intracellular epitope
pPDGFRβ	rb	sc-12909-R	Santa Cruz	0.8 µg ml^{-1}		recognizes phospho-Tyr 1021
p-Tyrosin	ms	pY20 / P4110	Sigma-Aldrich	2 µg ml^{-1}		
Stonin1	rb	86/85	home-made	30 µg ml^{-1}	30 µg ml^{-1}	purified[2]
Stonin2	rb	Stn2	home-made		1:500	purified[3], concentration not known
Vinculin	ms	hVIN-1 / V9131	Sigma	1:100	1:200	ascites, 8.6 mg/ml IgG1

IF: immunofluorescence; WB: western blotting; IP: immunoprecipitation; ms: mouse; rb: rabbit; gt: goat; gp: guinea pig; ha: Armenian hamster.

Secondary antibodies were either conjugated to horseradish peroxidase (HRP) for Western blot detection (GE Healthcare) or to Alexa-fluorophores in case of immunofluorescence experiments (Invitrogen).

Supplementary Table 2 Plasmids and siRNAs

insert	species	amino acids	mutations/ fusions	vector or sequence
Stonin1	mouse	full length	C-terminal HA	pcDNA3.1
Stonin1-N-term	mouse	1-404	C-terminal HA	pcDNA3.1
Stonin1-µHD	mouse	405-730	C-terminal HA	pcDNA3.1
Stonin1	mouse	full length	C-terminal tdTomato	ptdTomatoN1
Stonin1	mouse	full length	C-terminal EGFP	pEGFPN1
Stonin1	mouse	full-length	C-terminal EGFP	pRRLSIN.cPPT.PGK-GFP.WPRE (Addgene#12252) for lentiviral expression
Stonin1-µHD	mouse	405-730	C-terminal EGFP	pRRLSIN.cPPT.PGK-GFP.WPRE (Addgene#12252) for lentiviral expression
psPax2				lentiviral packaging plasmid (Addgene #12260)
pMD2.G				lentiviral packaging plasmid (Addgene #1259)
NG2	rat	full length	no tag	pcDNA; provided by W. Stallcup
NG2	rat	full length	internal EGFP after aa1312	pcDNA; subcloned from NG2 construct provided by W. Stallcup
NG2	rat	full length	internal SNAP tag after aa1312	pcDNA; subcloned from NG2 construct provided by W. Stallcup
NG2-tail1	rat	2256-2326	N-terminal GST	pGEX4T1
NG2-tail2	rat	2280-2326	N-terminal GST	pGEX4T1
NG2-tail3	rat	2305-2326	N-terminal GST	pGEX4T1
NG2-tail4	rat	2305-2322	N-terminal GST	pGEX4T1
Paxillin		full length	EGFP	gift from C. Ballestrem
Rab5	human	full length	N-terminal EGFP	pcDNA3.1
control siRNA	ms			GTAACTGTCGGCTCGTGG T
NG2 siRNA	ms			GGUCAAUCCUGUCAACG AU

Supplementary References

1. Schmidt, M.R. *et al.* Regulation of endosomal membrane traffic by a Gadkin/AP-1/kinesin KIF5 complex. *Proceedings of the National Academy of Sciences of the United States of America* **106**, 15344-15349 (2009).
2. Diril, M.K., Wienisch, M., Jung, N., Klingauf, J. & Haucke, V. Stonin 2 is an AP-2-dependent endocytic sorting adaptor for synaptotagmin internalization and recycling. *Developmental cell* **10**, 233-244 (2006).
3. Walther, K., Diril, M.K., Jung, N. & Haucke, V. Functional dissection of the interactions of stonin 2 with the adaptor complex AP-2 and synaptotagmin. *Proceedings of the National Academy of Sciences of the United States of America* **101**, 964-969 (2004).
